# Resistin and In-Hospital Mortality in Patients with Acute Ischemic Stroke: A Prospective Study

**DOI:** 10.3390/jcm13164889

**Published:** 2024-08-19

**Authors:** Ioana Cristina Bârsan, Silvina Iluţ, Nicoleta Tohănean, Raluca Pop, Ştefan Cristian Vesa, Lăcrămioara Perju-Dumbravă

**Affiliations:** 1 Faculty of Medicine, Iuliu Hațieganu University of Medicine and Pharmacy, 400012 Cluj-Napoca, Romania; ioana.barsan@umfcluj.ro; 2 Department of Neurosciences, Faculty of Medicine, Iuliu Hațieganu University of Medicine and Pharmacy, 400012 Cluj-Napoca, Romania; tohanean.nicoleta@umfcluj.ro (N.T.); lperjud@umfcluj.ro (L.P.-D.); 3 Department of Pharmacology, Toxicology and Clinical Pharmacology, Iuliu Haţieganu University of Medicine and Pharmacy, 400337 Cluj-Napoca, Romania; raluca.pop@umfcluj.ro (R.P.); stefan.vesa@umfcluj.ro (Ş.C.V.)

**Keywords:** acute ischemic stroke, leptin, resistin, in-hospital mortality

## Abstract

**Background/Objectives**: Understanding the prognostic factors of acute ischemic stroke (AIS) is essential for improving patient outcomes. The aim of this study was to establish the predictive role of plasmatic resistin and leptin on short-term mortality in adult patients with a first episode of AIS. **Methods**: This study enrolled 277 patients who were consecutively hospitalized for AIS. Demographic data, cardiovascular risk, comorbidities, and laboratory tests were collected. Death was noted if it occurred during hospitalization. **Results**: Death was recorded in 33 (11.9%) patients. Conducting multivariate analysis, the following variables were independent variables associated with in-hospital mortality: a resistin value of >11 ng/mL (OR 10.81 (95%CI 2.31;50.57), *p* = 0.002), a lesion volume of >18.8 mL (OR 4.87 (95%CI 1.87;12.67), *p* = 0.001), a NIHSS score of >7 (OR 5.88 (95%CI 2.01;17.16), *p* = 0.001), and the presence of IHD (OR 4.33 (95%CI 1.66;11.27), *p* = 0.003). This study has some limitations: single-center design (which may affect the generalizability of the results) and the potential impact of the COVID-19 pandemic on patient outcomes. **Conclusions**: This study demonstrated that resistin is a significant predictor of in-hospital mortality in AIS patients. Other established factors, such as a high NIHSS score, large lesion volume, and the presence of IHD, were reaffirmed as important predictors.

## 1. Introduction

Acute ischemic stroke (AIS) is an emergency condition that is responsible for sudden neurologic dysfunction caused by focal brain ischemia, confirmed by imaging evidence of acute infarction. On a worldwide scale, 87% of all stroke cases are ischemic. They represent the leading cause of both morbidity and mortality, with the greatest burden of this disease experienced by low- and middle-income countries [1,2]. The number of global deaths due to AIS increased from 2.04 million in 1990 to 3.29 million in 2019 and is anticipated to reach 4.90 million by 2030 [3]. Romania follows the same trend, with the highest incidence of AIS and top mortality rates in Europe [4]. Despite progress in acute stroke treatment, such as thrombolysis and endovascular interventions, the mortality rate for AIS remains elevated. This is particularly noticeable in elderly patients and those experiencing severe strokes [5].

Understanding the prognostic factors of AIS is essential for improving patient outcomes. Identifying predictors of intrahospital mortality in ischemic stroke patients is crucial, as it makes it possible for healthcare providers to deliver prompt and appropriate treatment. Among the numerous predictive factors that are known to increase stroke burden such as smoking, a high sodium diet, high blood arterial pressure, high low-density lipoprotein cholesterol, kidney dysfunction, high fasting plasma glucose, and high body mass index (BMI) or high National Institute of Health Stroke Scale (NIHSS) score, large lesion volume or comorbidity biomarkers like adipokines and, in particular, resistin and leptin, have become noticeable factors in AIS due to their roles in inflammation, metabolism, and cardiovascular health [3,6].

Resistin is a hormone that plays a significant role in metabolic and inflammatory processes. It is encoded by the RETN gene and was initially described as originating from the adipose tissue, which led to its classification as an adipokine [7]. After a major vascular event, such as AIS, resistin can induce a major inflammatory response, which can worsen neuronal injury and impede recovery. Resistin promotes inflammation by activating the production of pro-inflammatory cytokines, such as TNF-α, IL-6, and IL-12, and exacerbates atherosclerosis by increasing LDL cholesterol uptake in macrophages [8]. Resistin additionally upregulates the expression of several pro-inflammatory factors, including monocyte chemoattractant protein-1, endothelin-1, matrix metalloproteinases, and adhesion molecules that can lead to blood–brain barrier damage [9]. As the blood–brain barrier is affected by the inflammatory processes, immune cells infiltrate the area and exacerbate the local inflammation [10]. This will increase neural damage, disrupt even more of the blood–brain barrier, induce cerebral edema, and may increase the risk of a poor outcome. Leptin is another hormone that is secreted by adipocytes and has important functions in regulating energy balance, appetite, and metabolism. It also has pro-inflammatory properties. Low leptin levels were associated with worse outcomes and greater severity of stroke [11].

The aim of this study was to establish the predictive role of plasmatic resistin and leptin on short-term mortality in adult patients with a first episode of AIS.

## 2. Materials and Methods

A total of 277 patients were enrolled in this prospective, observational, analytical cohort study and were consecutively hospitalized with AIS in Cluj-Napoca Emergency County Clinical Hospital, Departments of Neurology I and II, between 1 December 2020 and 15 July 2021. The diagnosis was based on clinical assessment and native cranio-cerebral computed tomography (CT). This study was conducted in accordance with the Declaration of Helsinki and was approved by the Ethics Committee of the “Iuliu Hațieganu” University of Medicine and Pharmacy, Cluj-Napoca, Romania (protocol code no. 278 and date of approval 11 August 2020).

The inclusion criteria were the following: patients who were diagnosed with new AIS that were confirmed by imaging techniques, aged over 18 years, and with written informed consent obtained either from the patient or from family members. Exclusion criteria were hemorrhagic stroke, hemorrhagic transformation, patients diagnosed with COVID-19 infection, active neoplastic pathology, sepsis, documented autoimmune diseases, or those without confirmation by imaging assessment.

**Figure 1:**
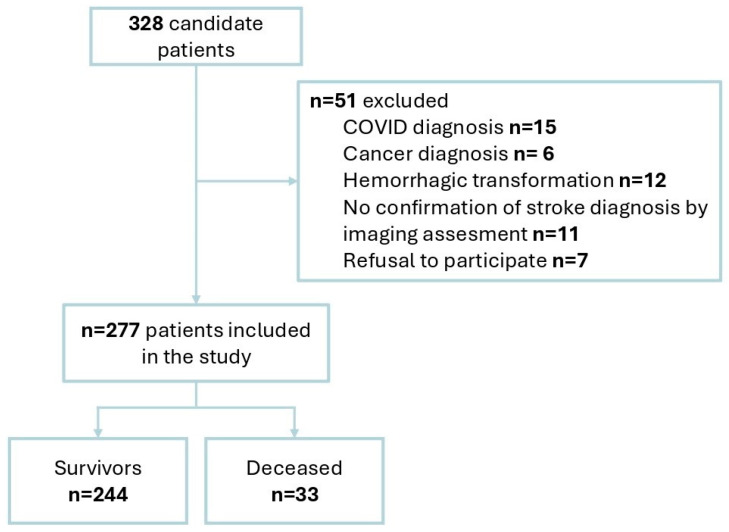
Flowchart of the patients included in the study.

Figure 1 presents the patients’ flow chart.

After admission, all patients had to answer detailed medical history questions, undergo neurological examination by a senior neurologist, be assessed using the NIHSS, and undergo general examination, including BMI scores. Demographic data (age, gender, and environment), cardiovascular risk factors (smoking, dyslipidemia, diabetes mellitus (DM), and arterial hypertension (AF)), comorbidities (atrial fibrillation (AF), heart failure (HF), ischemic heart disease (IHD), and valvulopathy), and laboratory tests (leptin, resistin, and CRP) were collected. Death was reported if it had occurred during hospitalization.

The presence of carotid plaques was reported during the ultrasound examination according to the following criteria: localized protrusion of the carotid wall, which was thicker than 1.5 mm or more than 50% of the intima-media thickness of the adjacent area [12]. AIS were classified by etiology using the Trial of Org 10172 in Acute Stroke Treatment (TOAST) classification in atherothrombotic, cardioembolic, small vessel disease, undetermined etiology, and other determined etiology [13]. Arterial territories were identified and classified into the medium cerebral artery (MCA), vertebrobasilar artery (VB), lacunar (L), and multiple territories. The ischemic lesion volume was calculated. The ellipsoid volume formula V= 4/3 π × (A/2) × (B/2) × (C/2) was applied to determine the lesion’s size. In this equation, A represents the longest axial diameter, B is the perpendicular diameter in the axial plane, and C is the craniocaudal diameter of the lesion.

The blood samples were collected on the first morning following admission in an EDTA tube and biochemistry tube with routine blood tests. Plasma samples were obtained from the EDTA collection tubes by centrifugation (10 min at 1000× *g*) within the first 30 min after collection. Aliquots of plasma were kept at −80 °C until analysis. The plasma resistin (code: RD191016100) and leptin (code: RD191001100) levels were quantified using commercially available ELISA kits (BioVendor R&D, Brno, Czech Republic). The protocols were performed following the instructions provided by the manufacturer. During the experimental procedure, absorbance readings and plate washing were conducted using an 800 TS ELISA microplate reader (Agilent Technologies Inc., Santa Clara, CA, USA) and a Biotek Microplate 50 TS washer (Agilent Technologies Inc., Santa Clara, CA, USA).

Both acute and prophylactic treatment approaches were considered. No patient was able to undergo a thrombectomy, as there was no technical platform in our center at the time. The possibility of transferring patients to a center equipped with a thrombectomy platform was hindered by the challenges posed by the COVID-19 pandemic.

Statistical analysis was performed using MedCalc^®^ Statistical Software version 22.021 (MedCalc Software Ltd., Ostend, Belgium; https://www.medcalc.org; 2024). The sample size was calculated from a pilot study (7 deceased and 35 survivors). The mean difference between the two groups for the resistin values was 9.53 ng/mL. For a type 1 (α) error of 0.1 and a type 2 (β) error of 0.05, we calculated a sample size of 34 patients in the deceased group and 170 patients in the survivor group. Qualitative data were characterized by frequency and percentage. Quantitative data were expressed as the median and 25th–75th percentiles, as the data distribution was non-normal according to the Shapiro–Wilk test. Comparisons between groups were performed using the Mann–Whitney test for the quantitative variables and chi-square tests for the qualitative variables. ROC analysis was used to establish a cut-off value for the association of several quantitative variables with death. The best cut-off threshold was chosen using the Youden index to ensure an optimal balance between sensitivity and specificity. Variables that were statistically significant in the univariate analysis were included in the multivariate logistic regression. The binary logistic regression analysis was employed to determine which variables were independently associated with mortality. Statistical significance was considered at a *p*-value of <0.003 after taking into account the Bonferroni correction for multiple comparisons.

## 3. Results

Death was recorded in 33 (11.9%) patients. Table 1 summarizes the demographic, clinical, imaging, and laboratory variables of survivors and patients who died during their hospital admission. Deceased patients were significantly older (*p* = 0.001), were more likely to have IHD (*p* < 0.001), and had higher NIHSS scores (*p* < 0.001). The resistin and CRP levels were significantly higher in those patients who died (*p* < 0.001). Most patients who died had an ACM stroke (*p* = 0.001). Additional variables can be found in Table S1.

Several of the cut-off values were calculated for the quantitative variables associated with death in the prior analysis (Table 2). Patients aged over 76 years, with an NIHSS score of >7, with resistin levels of >11 ng/mL, or with a lesion volume of >18.8 mL, were at a higher risk of dying during hospitalization.

To identify which of the variables were independently associated with death during hospitalization, we constructed a model using logistic regression (Table 3). Although ACM achieved a *p*-value of <0.05, a threshold of 0.003 was applied for statistical significance, provided by the Bonferroni correction, and thus, ACM was not independently associated with death. A resistin value of >11 ng/mL was the strongest independent variable associated with death, although it has a large 95% confidence interval (CI). A lesion volume of >18.8 mL, a NIHSS score of >7, and the presence of ischemic heart disease (IHD) were the other independent variables associated with in-hospital mortality.

## 4. Discussion

This present study evaluated several demographic, clinical, imagistic, and laboratory data as predictors of in-hospital mortality of patients with AIS. The results showed that age, the presence of IHD, NIHSS score, resistin, CRP, MCA territory, and lesion volume were associated with death in the univariate analysis. The multivariate analysis, however, showed that only high resistin values, large lesion volume, high NIHSS score, and IHD could be regarded as predictors of in-hospital mortality in patients with stroke. To our knowledge, this is the first study to show that resistin levels can predict in-hospital mortality in patients with AIS. The integration of resistin as a prognostic biomarker, together with several classic predictive markers such as the lesion volume, NIHSS score, or presence of comorbidities, could enhance the current protocols for managing AIS patients, allowing for a more precise risk stratification and personalized care. While further multi-center studies are needed to validate these findings, the evidence presented in our study suggests that resistin could be a valuable addition to the present predictive analysis.

Despite interventional therapies, acute ischemic stroke remains one of the main causes of disability and mortality (one in six deaths from cardiovascular diseases in the USA), especially in the elderly [14]. While various mechanisms contribute to the development of stroke, there are studies that show that inflammation plays a crucial role in many aspects of AIS, from the factors that increase the risk of onset up to poststroke outcome [15]. Inflammation is also a key factor in the development of atherosclerosis and the damage to brain tissue that can result from cerebral ischemia [16].

Plasma resistin levels are the most important variable associated with in-hospital mortality in this study. AIS determines a high degree of neuronal injury and an exacerbation of inflammatory processes [17]. Resistin, a prominent pro-inflammatory cytokine, has been described as implicated in the pathophysiology of AIS through several mechanisms [18]. High levels of resistin have been observed in patients with hypertension, diabetes mellitus, and other atherosclerotic diseases, which are known risk factors for AIS [19,20,21]. These diseases are characterized by chronic low-grade inflammation, and resistin exacerbates the inflammation by promoting the expression of pro-inflammatory cytokines and adhesion molecules [16]. Studies have shown that resistin levels rise after acute ischemic events, including stroke [21,22,23]. This increase may exacerbate the inflammatory cascade, further damaging the brain tissue. Resistin induces the infiltration of leukocytes into the ischemic brain areas, which can exacerbate local inflammation and worsen the disruption of the blood–brain barrier [24,25]. This can increase local edema neuronal damage and also impact recovery and rehabilitation outcomes. A study performed on elderly Finnish—the OPERA cohort—found that patients with a resistin level of >12.88 ng/mL had an increased mortality rate. Although the level of resistin is similar to our study, the OPERA cohort included an elderly population in general, not just those with stroke, and followed them for long periods of time (approximately six years) [26]. Similar to our study on AIS patients, the study by Kapłon-Cieślicka et al. found a threshold level of resistin (11.4 ng/mL) that is associated with increased mortality. While our study focuses on in-hospital mortality among AIS patients, the Kapłon-Cieślicka et al. study followed DM patients over a longer period (median follow-up of 5.4 years) and evaluated the long-term mortality risk [27]. In a small prospective study on AIS patients, Bouziana et al. did not find that the resistin or leptin levels differed between patients who died during hospitalization and those who were discharged, although the levels were higher in deceased patients. The number of observed deaths was small, which might explain the lack of statistical power in detecting significant differences between groups [28]. In contrast, our study demonstrated that resistin is very closely related to in-hospital mortality. The discrepancies between the Bouziana et al. study and our own could be attributed to the differences in study design, sample sizes (much larger in our study), or population characteristics. Another study showed that high resistin levels were found in patients who died 30 days, 1 year, and 5 years after AIS [29]. By comparison, our study investigated the mortality rate during the in-hospital period, which is often the most vulnerable time for AIS patients. By concentrating on the acute phase, our research provides critical insights into the immediate prognostic value of resistin as a significant predictor of in-hospital mortality. Lee at al. evaluated the association of resistin with short-term unfavorable functional outcome, but not with mortality, as our research did [22]. In contrast to our findings, a recent meta-analysis by Agbaedeng et al. explored the association between various adipokines and mortality at 6 months in patients with AIS and found that adipokines such as fatty acid-binding protein-4 and visfatin were significantly associated with the outcome [17]. However, resistin was not found to be associated with mortality in their analysis, primarily due to the lack of sufficient studies on resistin. This highlights the importance and novelty of our study, which specifically investigates resistin and establishes its significant role as a predictor of in-hospital mortality in AIS patients. Resistin could be added to the routine biochemical markers measured in AIS patients, as it may help physicians better identify those at higher risk of in-hospital mortality, enabling more tailored interventions and closer monitoring.

In this present study, patients with AIS who died during hospitalization have a median NIHSS score of 14. Further analysis showed that in the case of a patient with an NIHSS score greater than 7, the probability of dying during admission is six times higher. Since it was introduced in clinical practice, the NIHSS has proven to be very useful as a solid predictor of stroke patients’ improvement or decline. The NIHSS is a valuable predictor of poststroke outcomes because it provides an extensive, comprehensive evaluation of brain functions, including consciousness, vision, sensation, movement, speech, and language. It has been used in numerous studies that examined the short- and long-term outcomes of these patients, and our study adds to the existing body of medical literature [6,30,31,32,33,34].

A large lesion volume is another strong predictor of in-hospital mortality in the present study. The volume of the brain lesion that results from an ischemic stroke can be directly linked to the severity of the disease [35]. Several studies have revealed that a large lesion volume is an important predictor of death after a stroke, which supports our findings [6,36,37,38]. Larger lesions usually indicate a higher chance of extensive brain damage due to increased intracranial pressure and hemorrhagic transformation and are associated with higher mortality rates. The neuroinflammatory response induces a series of alterations, such as blood–brain barrier damage, localized edema, the appearance of reactive oxygen species, and other markers of oxidative stress, which seem to be associated with the size of the lesion [39,40].

IHD was present in twice as many patients who died compared to those who survived. IHD and stroke share several common risk factors, including AH, DM, dyslipidemia, smoking, and obesity, and IHD is a risk factor for stroke by itself [41]. These factors contribute to the development of atherosclerosis, and when IHD and stroke coexist, they signal the presence of extensive atherosclerosis [42,43]. IHD can lead to complications such as HF, arrhythmias, or other cardiovascular events, which can lead to an increased mortality risk. Although both HF and AF were more frequent in deceased patients, the difference was not statistically significant in our study, and that means that there are other possible factors through which IHD increases mortality in stroke. The presence of IHD can exacerbate systemic inflammation. Additionally, the peripheral immune response to stroke begins quickly, with the release of immunoactive molecules or activated immune cells triggering widespread cytokine release into the bloodstream, which will contribute to poorer outcomes after a stroke [43]. Moreover, patients with IHD may have a reduced cardiovascular reserve, which makes them more vulnerable to a major event like a stroke. There are several studies showing that IHD is associated with an increased risk of short-term mortality after stroke, just like our research [44,45,46].

Age and MCA stroke were variables associated with death in the univariate analysis but were not independent predictors of stroke mortality in the multivariate analysis. The relationship between age or MCA stroke and mortality is likely influenced by other factors. Advanced age, in our study, of >76 years, can be associated with an increased risk of death after stroke, mainly through the presence of important comorbidities (IHD, for example) that can reduce its statistical value as a predictor. The elderly might also exhibit frailty and are more likely to develop complications during hospitalization (infection or venous/pulmonary thromboembolism) [47]. Aggressive preventive strategies, including stricter management of vascular risk factors, such as AH, DM, and hyperlipidemia, have been proven to lower both the incidence and mortality rates of ischemic stroke and must be applied in the elderly with a history of stroke [48]. MCA stroke was an important predictive factor for mortality in our study; however, due to the correction for multiple comparisons, it did not reach the statistical significance threshold in multivariate regression, although a clear trend was observed. The fact that the severity of stroke and lesion volume were independent predictors might overshadow the importance of the area affected by stroke. Some studies found that MCA stroke is associated with mortality after stroke [49,50]. Our findings align with the medical literature, and in a larger study, age and MCA stroke could emerge as independent predictors of death in AIS patients.

The leptin levels were not statistically significantly associated with mortality in the present study. Low levels of leptin were determined in the deceased group, which is in accordance with the literature [11]. The analysis of the Framingham study and a study by Dai et al. did not find leptin to be associated with the risk of stroke [51,52]. Our results contribute to the body of contradictory literature on the subject of leptin and stroke. A definitive answer may be provided by future experimental studies on this topic.

The study was conducted during the first two peaks of the COVID-19 pandemic, which significantly affected the management of stroke patients [53]. Access to care for non-COVID-19 cases was limited, as resources were heavily redirected to managing the pandemic. This resulted in fewer stroke-related admissions, more severe cases due to delays in transport, imaging procedures, and diagnosis, and a noticeable drop in thrombolysis rates [54]. These factors likely contributed to the variations experienced in the standard of care provided during the study period, potentially impacting patient outcomes [55]. The context of the COVID-19 pandemic should be carefully considered when interpreting the results, as it may have significantly affected the management and outcomes of stroke patients.

Several strengths of the study include its prospective nature, an adequate number of patients that enhances statistical significance, and is the first study to prove that resistin can predict in-hospital mortality after stroke.

### 4.1. Limitations

Some study limitations might be indicated by the fact that this study was conducted in a single center, even if it is a tertiary one, and it enrolled patients during the COVID-19 pandemic, which may have impacted patient management and healthcare resources. While the study was conducted in a tertiary care center, the results may not be fully applicable to other settings with different stroke management protocols. The single-center design also reduces the analysis of possible variations in practice care and outcomes that might be present in a multi-center study. The results should be interpreted with caution when considering their application to broader populations. This study’s design is observational, which makes it difficult to establish a direct causal relationship between the resistin levels and patient outcomes. In our study, while significant associations were identified between elevated resistin levels and increased in-hospital mortality among AIS patients, these findings cannot be interpreted as evidence that resistin directly causes poorer outcomes. Resistin is involved in the exacerbation of inflammatory processes, but the specific mechanisms by which it could exacerbate brain injury, influence the blood–brain barrier, or affect recovery in stroke patients cannot be definitively proved by an observational study. Experimental studies are needed to investigate the molecular and cellular pathways involved. The 95% CIs for resistin in relation to its association with in-hospital mortality were notably large, indicating a high degree of variability in the data. Although we found a statistically significant association, the precision of the findings is limited. This could be due to the relatively small number of deaths observed in the study, which can result in less precise data. Also, the resistin levels showed a wide range of variability among patients, possibly due to individual differences in inflammatory response and comorbidities, further contributing to the large CIs. These large CIs suggest that the results should be interpreted with caution, particularly regarding the strength and accuracy of the association between resistin and patient outcomes.

### 4.2. Future Directions

Resistin could be added to the routine biochemical markers measured in AIS patients, as it may help physicians better identify those at higher risk of in-hospital mortality, enabling more tailored interventions and closer monitoring. Future studies should focus on expanding the research in multi-center settings and on investigating the long-term impact of resistin levels on AIS outcomes over 1 to 5 years. These studies should include a broader range of adipokines and other pro-inflammatory markers to establish the most reliable predictor. Additionally, the potential role of resistin as a predictor of functional recovery should be explored. The mechanisms by which resistin is associated with mortality should be investigated through experimental research. Finally, therapeutic approaches aimed at reducing inflammation in patients with high resistin levels should be explored in randomized controlled trials, including the use of statins alone or in combination with other lipid-lowering drugs, colchicine, or anti-cytokine agents.

## 5. Conclusions

Our study suggests that elevated resistin levels may be significantly associated with an increased risk of in-hospital mortality in patients with AIS. Additionally, established factors such as a high NIHSS score, large lesion volume, and the presence of ischemic heart disease were reaffirmed as important predictors of in-hospital mortality. However, given the observational nature of our study and the single-center design, further multi-center studies are necessary to validate these results and to explore the underlying mechanisms by which resistin may influence in-hospital outcomes in AIS patients.

## Figures and Tables

**Table 1:** Comparison of group characteristics between survivors and deceased patients.

Variables		Survivors (*n* = 244)	Deceased (*n* = 33)	*p*-Value
Age (years) *		72 (62.5; 80)	81 (77; 84)	0.001
Sex	F	129 (52.9%)	16 (48.5%)	0.7
	B	115 (47.1%)	17 (51.5%)	
Environment	Rural	77 (31.6%)	15 (45.5%)	0.1
	Urban	167 (68.4%)	18 (54.5%)	
BMI (kg/m^2^) *		27.5 (24.4; 30.9)	27.5 (26.1; 31.2)	0.4
Smoking	No	200 (82%)	30 (90.9%)	0.32
	Yes	44 (18%)	3 (9.1%)	
AH	No	40 (20.1%)	6 (18.2%)	0.9
	Yes	195 (79.9%)	27 (81.8%)	
AF	No	193 (79.1%)	23 (69.7%)	0.3
	Yes	51 (20.9%)	10 (30.3%)	
HF	No	218 (89.3%)	28 (84.8%)	0.3
	Yes	26 (10.7%)	5 (15.2%)	
IHD	No	199 (81.6%)	17 (51.5%)	<0.001
	Yes	45 (18.4%)	16 (48.5%)	
Valvulopathy	No	210 (86.1%)	29 (87.9%)	1
	Yes	34 (13.9%)	4 (12.1%)	
DM	No	180 (73.8%)	20 (60.6%)	0.1
	Yes	64 (26.2%)	13 (39.4%)	
Dyslipidemia	No	58 (23.8%)	9 (27.3%)	0.8
	Yes	186 (76.2%)	24 (72.7%)	
NIHSS score *		6 (3; 10)	14 (8; 21)	<0.001
Leptin (ng/mL) *		46.8 (18.1; 88.5)	35.9 (17.3; 87.7)	0.2
Resistin (ng/mL) *		12.5 (8.8; 20.5)	22.3 (13.9; 31.9)	<0.001
CRP (mg/L) *		1 (0.3; 2.7)	3.6 (1.6; 9.5)	<0.001
MCA stroke	No	104 (42.6%)	4 (12.1%)	0.001
	Yes	140 (57.4%)	29 (87.9%)	
Vascular territories	VB	56 (23%)	3 (9.1%)	0.01
	MCA	133 (54.4%)	28 (84.8%)	
	L	34 (13.9%)	1 (3%)	
	Multiple	21 (8.6%)	1 (3%)	
Lesion volume (mL) *		12.2 (6.2; 29.5)	32.9 (16.5; 39.5)	<0.001
Carotid plaque	No	63 (25.8%)	11 (33.3%)	0.4
	Yes	181 (74.2%)	22 (66.7%)	
Thrombolysis	No	200 (82%)	28 (84.6%)	0.8
	Yes	44 (18%)	5 (15.2%)	

* Median (25–75 percentiles); *n*: number of cases; BMI: body mass index; AH: arterial hypertension; AF: atrial fibrillation; HF: heart failure; IHD: ischemic heart disease; DM: diabetes mellitus; NIHSS: National Institute of Health Stroke Scale; CRP: C-reactive protein; MCA: middle cerebral artery; VB: vertebrobasilar; L: lacunar.

**Table 2:** AUCs for variables regarding mortality.

Variables	AUC (95% CI)	Cut-Off	Sensitivity (95% CI)	Specificity (95% CI)	*p*
Age	0.796 (0.745–0.843)	>76 years	72.60 (60.9–82.4)	76.6 (69.5–82.7)	<0.001
NIHSS	0.787 (0.734–0.833)	>7	81.8 (64.5–93.0)	61.4 (55.1–67.6)	<0.001
Resistin	0.716 (0.659–0.769)	>11 ng/mL	93.9 (79.8–99.3)	42.2 (35.9–48.7)	<0.001
CRP	0.751 (0.690–0.805)	>1.1 mg/L	90 (73.5–97.9)	55.3 (48.3–62.3)	<0.001
Lesion volume	0.742 (0.686–0.792)	>18.8 mL	72.73 (54.5–86.7)	66.8 (60.5–72.7)	<0.001

NIHSS: National Institute of Health Stroke Scale; CRP: C-reactive protein.

**Table 3:** Multivariate analysis for in-hospital death.

Variables	B	*p*	OR	95% CI for OR	
				Min	Max
Age > 76 years	0.913	0.071	2.491	0.925	6.710
IHD	1.467	0.003	4.334	1.667	11.271
NIHSS > 7	1.773	0.001	5.887	2.019	17.164
Resistin > 11 ng/mL	2.381	0.002	10.812	2.312	50.570
MCA stroke	1.446	0.022	4.245	1.230	14.653
Lesion volume > 18.8 mL	1.584	0.001	4.875	1.876	12.671
Constant	−3.203	<0.001	0.041		

IHD: ischemic heart disease; NIHSS: National Institute of Health Stroke Scale; middle cerebral artery.

## Data Availability

The datasets presented in this article are not readily available because the data are part of an ongoing study.

## Supplementary Materials

The following supporting information can be downloaded at: https://www.mdpi.com/article/10.3390/jcm13164889/s1, Table S1: Comparison between survivor and deceased.
