# Supplementary material for: Resistin and In-Hospital Mortality in Patients with Acute Ischemic Stroke: A Prospective Study
Source: J Clin Med. 2024 Aug 19;13(16):4889. doi: 10.3390/jcm13164889 (PMC11355181; doi:10.3390/jcm13164889)
Supplement: Supplementary file 1 [file jcm-13-04889-s001.zip › jcm-3170572-supplementary.pdf]

Table S1. Supplemental. Comparison between survivor and deceased.

Variables		Survivor (n=244)	Deceased (n=33)	p
History of stroke	No	178 (73%)	21 (63.6%)	0.3
	Yes	66 (27%)	12 (36.4%)	
History of cancer	No	226 (92.6%)	32 (97%)	0.7
	Yes	18 (7.4%)	1 (3%)	
History of upper gastrointestinal bleeding	No	241 (98.8%)	33 (100%)	1
	Yes	3 (1.2%)	-	
Chronic obstructive pulmonary disease	No	232 (95.1%)	27 (81.8%)	0.01
	Yes	12 (4.9%)	6 (18.2%)	
Gout	No	229 (93.9%)	32 (97%)	0.7
	Yes	15 (6.1%)	1 (3%)	
Hypothyroidism	No	228 (93.4%)	33 (100%)	0.2
	Yes	16 (6.6%)	-	
Peripheral arterial disease	No	239 (98%)	32 (97%)	0.5
	Yes	5 (2%)	1 (3%)	
Metabolic syndrome	No	96 (40.1%)	13 (40.6%)	1
	Yes	142 (59.9%)	19 (59.4%)	
Anemia	No	220 (82%)	27 (81.8%)	1
	Yes	44 (18%)	6 (18.2%)	
Bronchial asthma	No	232 (95.1%)	32 (97%)	1
	Yes	12 (4.9%)	1 (3%)	
Chronic kidney disease	No	232 (95.1%)	29 (87.9%)	0.1
	Yes	12 (4.9%)	4 (12.1%)	
Liver cirrhosis	No	243 (99.6%)	33 (100%)	1
	Yes	1 (0.4%)	-	
Depression	No	221 (90.6%)	33 (100%)	0.08
	Yes	23 (9.4%)	-	
Alcohol consumption	No	221 (90.6%)	30 (90.9%)	1
	Yes	23 (9.4%)	3 (9.1%)	
Waist circumference (cm)*		100 (83;118)	101 (80.5;110.5)	0.7
Total cholesterol (mg/dL)*		170.5 (138.2;199)	151.5 (122.2;189)	0.1
HDL cholesterol (mg/dL)*		45 (37;53)	43 (33;53.7)	0.6
LDL cholesterol (mg/dL)*		102 (74.2;125.7)	92.5 (60; 117.5)	0.1
Triglycerides (mg/dL)*		114 (84;160)	85 (70.5;137.5)	0.01
SBP (mmHg)*		165 (150;180)	167 (150;190)	0.2
DBP (mmHg)*		91.5 (80;100)	95 (87;106)	0.1
HR (bpm)*		90 (83;103)	100.5 (83;119.2)	0.08
Hemoglobin (g/dL)*		15 (13.9;16.2)	14.9 (13.3;16.4)	0.2
Trombocytes (x 109/L)*		274 (218.5;331.5)	293.5 (220;344)	0.8
AST (IU/L)*		31 (24;48.5)	48 (32;141.2)	0.005
ALT (IU/L)*		29 (19;38)	25(18;52)	0.9
GGT (IU/L)*		29.5 (17;52)	27 (17;50)	
Creatinine (mg/dL)*		1.03 (0.83; 1.36)	1.36 (0.97;2.35)	0.02

* median (25-75 percentiles); n: number of cases; AST - aspartate aminotransferase; ALT - Alanine transaminase; GGT - gamma-glutamyl transferase.
